# Supplementary figures and images for: Population coding strategies in human tactile afferents
Source: PLoS Comput Biol. 2022 Dec 7;18(12):e1010763. doi: 10.1371/journal.pcbi.1010763 (PMC9762576; doi:10.1371/journal.pcbi.1010763)

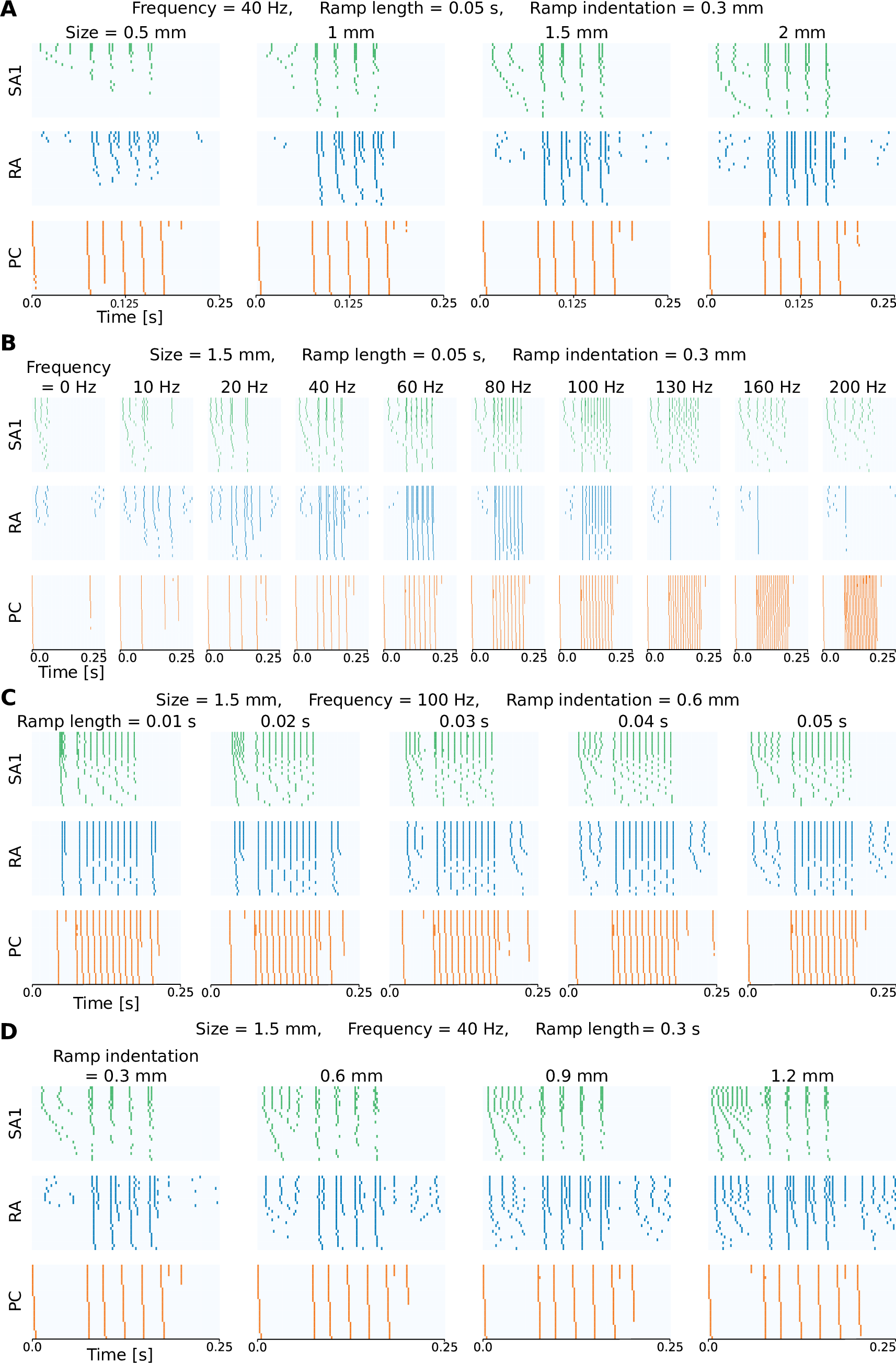

Supplement: S1 Fig — Responses are shown for the three afferent classes as a function of (A) stimulus size, (B) frequency, (C) ramp length, and (D) ramp amplitude. Note that we have conditioned on the remaining features for each panel and that the afferent densities chosen in this example correspond to the ones in the finger. (TIF) [file pcbi.1010763.s001.tif]

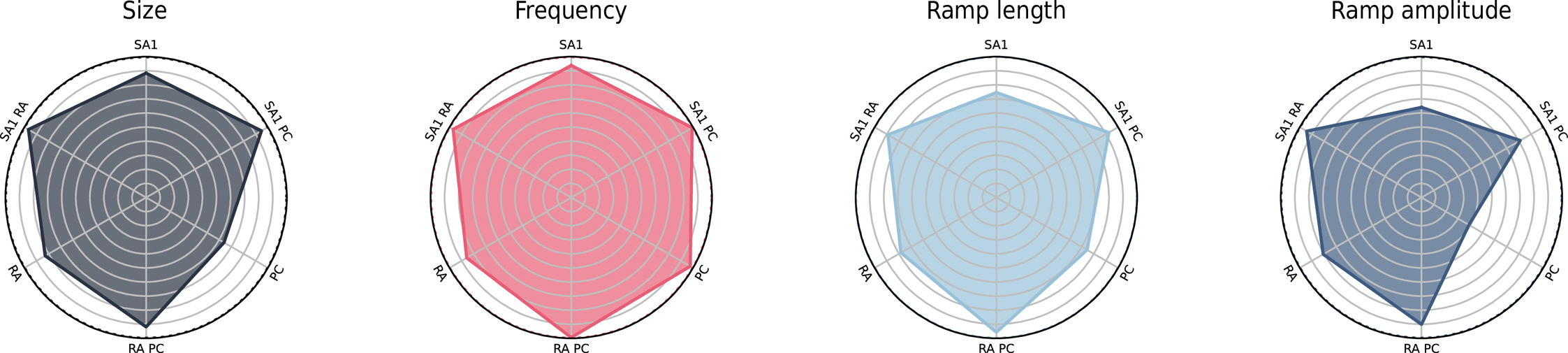

Supplement: S2 Fig — The analysis presented in Fig 5A (panel iv) was redone using a spatial NMF (rather than the spatiotemporal version) to test the robustness of the results. While this analysis extracts somewhat different information values, the main result that afferent classes working together increase the overall information content is preserved. (TIF) [file pcbi.1010763.s002.tif]
